# Concentration-driven structural evolution and enhanced photoluminescence dynamics in Ho^3+^-activated SrMoO_4_ nanophosphors

**DOI:** 10.1039/d6ra05779a

**Published:** 2026-10-12

**Authors:** Arpitha H J, Kartik Gopal, Sunitha D V, D. S. Prem Kumar, Kalappa Prashantha

**Affiliations:** a Department of Physics, School of Applied Sciences, REVA University Bengaluru-560064 Karnataka India sunitha.dv@reva.edu.in +91-7760884884; b Center for Advance Materials and Research in Physics, REVA Research Center, REVA University Bengaluru 560064 Karnataka India; c Department of Physics, Sri Adichunchanagiri First Grade College Channarayapatna, Hassan District-573116 Karnataka India; d Centre for Quality Assurance and Non-Destructive Evaluation (CQNDE), Sathyabama Institute of Science and Technology Chennai 600119 India; e ACU Centre for Research and innovation, Adichunchanagiri University Mandya District-571448 Karnataka India

## Abstract

Rare-earth-activated molybdate phosphors have attracted considerable interest because of their tunable structural and luminescence properties for photonic applications. In this work, Ho^3+^-doped SrMoO_4_ nanoparticles were synthesized through a controlled hydrothermal method employing Chikku (*Manilkara zapota*) leaf extract as a plant-derived processing additive. Structural characterization revealed the formation of a phase-pure tetragonal scheelite SrMoO_4_ structure for all investigated Ho^3+^ concentrations, with no detectable secondary crystalline phases. The observed structural changes are consistent with Ho^3+^ incorporation into the SrMoO_4_ host lattice, although direct confirmation of the substitutional site requires complementary characterization. The average crystallite size decreased from approximately 34 to 25 nm with increasing Ho^3+^ concentration from 1 to 7 mol%. UV-vis diffuse reflectance measurements showed a gradual modification of the absorption-edge profile, with the estimated optical band gap increasing from 4.10 to 4.17 eV. Under 451 nm excitation, the samples exhibited characteristic visible Ho^3+^ emissions, with prominent green and red emission bands near 545 and 658 nm, respectively. The relative emission intensity increased with Ho^3+^ concentration, reaching the highest value for the 7 mol% sample. Time-resolved photoluminescence measurements revealed a progressive increase in the emission lifetime from 148.62 µs for pristine SrMoO_4_ to 326.95 µs for the 7 mol% Ho^3+^-doped sample. No reduction in lifetime was observed within the investigated concentration range, indicating that significant concentration quenching is not evident up to 7 mol% Ho^3+^. Overall, the results demonstrate a concentration-dependent relationship between the structural characteristics, morphology, visible photoluminescence, and emission decay dynamics of Ho^3+^ activated SrMoO_4_ nanoparticles prepared through a plant-assisted hydrothermal route.

## Abstract 2

Ho^3+^ concentration enhances the visible photoluminescence of SrMoO_4_ nanoparticles and progressively extends the emission lifetime from 148.62 to 326.95 µs.

## Graphical abstract

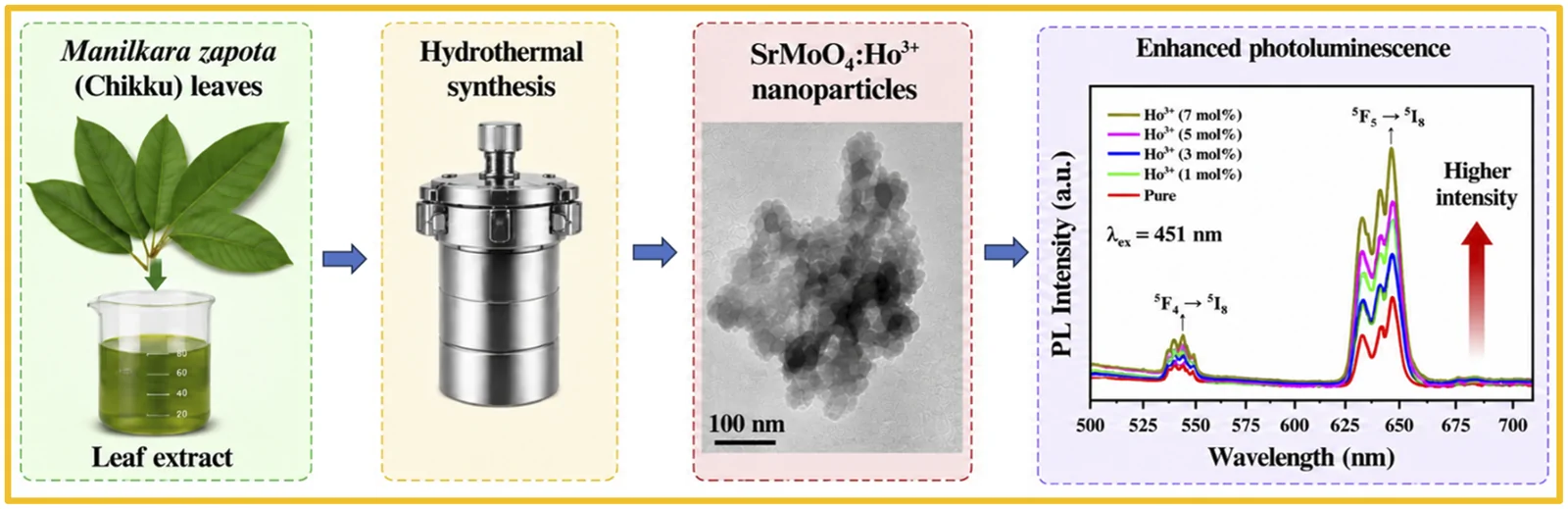

## 1. Introduction

Rare-earth activated luminescent materials have become central to the advancement of modern photonic technologies owing to their distinctive optical behavior, including sharp emission bands, long radiative lifetimes, and high resistance to photochemical degradation.^1–3^ These characteristics arise from parity-forbidden intra-configurational transitions within partially shielded 4f orbitals, which are only weakly influenced by the surrounding lattice environment.^4,5^ As a result, rare-earth ions can sustain stable emission under continuous excitation, making them indispensable in applications ranging from solid-state lighting,^6^ and flat-panel displays^7^ to optical sensing,^8^ laser systems,^9^ and anti-counterfeiting technologies.^10^ In recent years, the increasing demand for energy-efficient light sources and color-tunable phosphors has driven intensive research into host-activator combinations capable of delivering high emission efficiency, thermal stability, and long-term operational reliability.^11,12^ Among the various strategies for improving luminescent performance, the selection of an appropriate host lattice plays a decisive role in determining emission intensity, spectral distribution, and recombination dynamics. Oxide-based hosts such as aluminates,^13^ silicates,^14^ phosphates,^15^ and tungstates,^16^ have been widely explored due to their chemical robustness and structural flexibility. For example, aluminate hosts such as SrAl_2_O_4_ (ref. 17) and CaAl_2_O_4_ (ref. 18) have demonstrated strong persistent luminescence, particularly when activated with Eu^2+^ and Dy^3+^ ions, owing to their ability to support deep trap states that store excitation energy for extended durations. Similarly, silicate-based phosphors, including Y_2_SiO_5_ (ref. 19) and Ba_2_SiO_4_,^20^ have been investigated for their high thermal stability and compatibility with high-power light-emitting diode systems. Despite these advantages, many silicate and aluminate systems suffer from limitations such as high synthesis temperatures, complex defect chemistry, and sensitivity to moisture, which can adversely affect emission stability during prolonged operation.

Phosphate and tungstate compounds have also attracted attention as alternative host materials due to their relatively low phonon energies and efficient energy transfer characteristics. For instance, phosphate hosts such as LaPO_4_ (ref. 21) and YPO_4_ (ref. 22) exhibit excellent optical transparency and structural stability, making them suitable for rare-earth doping in luminescent and laser applications. Tungstate-based materials, including CaWO_4_ (ref. 23) and SrWO_4_,^24^ have demonstrated strong emission efficiency under ultraviolet excitation and have been widely studied for their scintillation and luminescence properties. However, these systems often exhibit limited flexibility in tuning emission color without introducing additional dopants or structural modifications, which can complicate material design and processing.

In comparison with these host families, molybdate compounds have emerged as particularly promising candidates for advanced luminescent applications due to their favorable electronic structure, structural stability, and adaptability to rare-earth substitution.^25^ The presence of tetrahedral MoO_4_^2−^ units within the lattice contributes to strong optical absorption and efficient energy transfer to activator ions. Furthermore, the relatively low phonon energy of molybdate frameworks reduces non-radiative relaxation processes,^26^ thereby enhancing radiative recombination efficiency.^27^ These features make molybdate hosts attractive for developing phosphor materials capable of sustaining stable emission across a broad spectral range. Strontium molybdate (SrMoO_4_), which crystallizes in a tetragonal scheelite structure,^28^ has gained increasing attention as a versatile host lattice for rare-earth ions. The scheelite structure provides crystallographic sites that can accommodate rare-earth ions; however, the incorporation of trivalent Ho^3+^ ions at divalent Sr^2+^ sites requires charge compensation and cannot be established conclusively from XRD alone. In the present study, the observed changes in lattice parameters and the absence of detectable impurity phases are considered consistent with Ho^3+^ incorporation into the SrMoO_4_ lattice; however, direct confirmation of the local chemical state and site occupancy would require complementary spectroscopic characterization. Rare-earth ions such as Eu^3+^, Tb^3+^, and Dy^3+^ can produce intense red, green, and yellow emissions,^29–32^ respectively, when incorporated into molybdate hosts. These findings highlight the potential of scheelite-type compounds as tunable phosphor materials for color-specific emission applications.

Despite these promising characteristics, the relationship between dopant concentration and emission dynamics in rare-earth activated molybdate systems remains an area of ongoing investigation. In particular, the mechanisms governing energy transfer, concentration-dependent recombination behavior, and emission stability in Ho^3+^-activated molybdate lattices have not been comprehensively explored. Holmium ions possess a complex energy level structure with multiple transitions spanning the visible region, allowing generation of diverse emission colors depending on the excitation wavelength and local crystal field environment.^33,34^ The presence of closely spaced energy levels also facilitates cross-relaxation and energy migration processes, which can significantly influence luminescence efficiency at higher dopant concentrations. A key factor controlling luminescent performance in rare-earth doped materials is the balance between radiative and non-radiative recombination pathways. When activator ions are introduced into the host lattice, they create a discrete energy levels that can temporarily trap excited electrons before releasing energy in the form of photons. At low dopant concentrations, these ions are typically well separated, allowing efficient radiative transitions with minimal interaction between neighboring centers. As the concentration increases, the distance between activator ions decreases, enabling energy transfer interactions that may either enhance emission through cooperative processes or reduce efficiency through concentration quenching. Understanding this concentration-dependent behavior is essential for identifying the optimal dopant level that maximizes luminescence performance. Time-resolved photoluminescence analysis provides valuable insight into these processes by measuring the lifetime of excited states within the material.^35,36^ The decay lifetime reflects the average duration that an electron remains in an excited state before recombining, serving as an indicator of the efficiency of radiative emission. Longer lifetimes generally suggest improved energy confinement and reduced non-radiative losses, while shorter lifetimes may indicate rapid recombination or increased interaction between luminescent centers. By correlating lifetime measurements with structural parameters such as crystallite size and lattice distortion, researchers can gain a deeper understanding of the mechanisms responsible for enhanced emission behavior. Recent advances in nanostructured materials have further highlighted the importance of particle size and morphology in determining optical properties. Reduction in crystallite size can influence the distribution of surface defects, modify electronic band structure, and alter light absorption behavior. In nanoscale phosphors, these effects often lead to changes in emission intensity and spectral distribution. Additionally, morphological evolution from spherical to anisotropic structures can affect light scattering and emission efficiency, emphasizing the need for precise control over synthesis conditions during material preparation. Although several studies have investigated rare-earth doped molybdate phosphors, systematic evaluation of Ho^3+^ incorporation in SrMoO_4_, particularly with respect to concentration-dependent structural modification and luminescence dynamics, remains limited. Most existing reports focus primarily on emission intensity without establishing a comprehensive relationship between dopant concentration, crystal structure, and recombination behavior. A detailed understanding of these interrelated factors is necessary for designing phosphor materials with predictable optical performance and stable emission characteristics.

However, a systematic correlation among Ho^3+^ concentration, structural evolution, morphology, visible emission characteristics, and time-resolved luminescence behavior in Ho^3+^-activated SrMoO_4_ remains comparatively limited. The present study addresses this aspect by investigating a series of Ho^3+^ concentrations under identical hydrothermal processing conditions and correlating the resulting crystallite-size variation and morphological evolution with photoluminescence intensity, emission characteristics, and decay lifetime. The use of Chikku (*Manilkara zapota*) leaf extract as a plant-derived processing additive further provides a plant-assisted synthesis route for this systematic concentration-dependent investigation. Since a direct extract-free or conventional-surfactant control was not performed, the present work does not claim that the plant extract provides superior structural or luminescence properties; rather, its use constitutes the synthesis approach adopted for examining the concentration-dependent behavior of Ho^3+^-activated SrMoO_4_.

Plant-derived extracts have attracted increasing interest as processing additives in nanoparticle synthesis because their naturally occurring organic constituents can interact with precursor species and particle surfaces during nucleation and growth. In particular, *Manilkara zapota* (Chikku/Sapodilla) leaf extract has previously been employed in the preparation of metal nanoparticles, including Ag and Au nanoparticles, demonstrating its applicability in plant-assisted nanomaterial synthesis.^37,38^ Previous studies have also reported phenolic, flavonoid, and other organic constituents in *M. zapota* leaf extract, which may provide functional groups capable of interacting with inorganic species and nanoparticle surfaces.^39–41^ Based on this literature precedent, Chikku leaf extract was selected in the present study as a plant-derived processing additive during the hydrothermal synthesis of SrMoO_4_:Ho^3+^ nanoparticles. However, the detailed phytochemical composition and surface-active properties of the specific extract used in the present work were not independently characterized. Therefore, the present study does not assign the observed structural or morphological characteristics to a specific phytochemical constituent or claim a specific surfactant mechanism. Recent studies on rare-earth-activated scheelite and molybdate systems further demonstrate the diversity of structural, optical, and application-oriented investigations in this material family. Ho^3+^-doped SrMoO_4_ has previously been investigated, particularly in combination with Dy^3+^, with emphasis on temperature-dependent luminescence, luminescence thermometry, and lighting applications.^42^ In related scheelite systems, Eu^3+^-activated SrWO_4_ has been studied mainly for its structural, morphological, and red-emission characteristics,^43^ while Yb^3+^/Nd^3+^-doped CaMoO_4_ has been reported for rapid synthesis and near-infrared photoluminescence.^44^ These studies confirm the broad potential of rare-earth-activated scheelite-type molybdate and tungstate hosts, but their primary objectives and activator systems differ from those of the present work. In contrast, the present study systematically examines the effect of Ho^3+^ concentration on the structural evolution, morphology, visible photoluminescence, and time-resolved emission behavior of SrMoO_4_ under identical hydrothermal processing conditions. The core contribution is therefore the concentration-dependent correlation of structural and morphological changes with visible emission characteristics and decay lifetime, rather than the simple observation of enhanced photoluminescence or prolonged lifetime following rare-earth activation.

In the present work, a series of Ho^3+^-activated SrMoO_4_ nanoparticles with varying dopant concentrations were synthesized using a controlled hydrothermal method employing Chikku (*Manilkara zapota*) leaf extract as a plant-derived processing additive. The influence of Ho^3+^ concentration on structural parameters, optical absorption behavior, emission characteristics, morphology, and time-resolved photoluminescence was systematically investigated. The study particularly focuses on correlating concentration-dependent structural and morphological changes with visible emission intensity and decay lifetime. The resulting relationships provide a systematic assessment of the concentration-dependent luminescence behavior of Ho^3+^-activated SrMoO_4_ prepared through a plant-assisted hydrothermal route.

## 2. Experimental methods

### 2.1. Materials

Strontium nitrate hexahydrate [Sr(NO_3_)_2_·6H_2_O], ammonium heptamolybdate tetrahydrate [(NH_4_)_6_Mo_7_O_24_⋅4H_2_O], holmium nitrate pentahydrate [Ho(NO_3_)_3_·5H_2_O], and sodium hydroxide (NaOH) were procured from SD Fine Chemicals Ltd, Bangalore, India. A fresh Chikku (Sapodilla) leaf sample was collected from Channarayapatna, Hassan District, Karnataka, India (12.90°N 76.39°E). All chemicals were of analytical grade and were used without further purification.

### 2.2. Methods

#### 2.2.1 Preparation of Chikku leaf extract

Freshly collected Chikku leaves were thoroughly washed with tap water 5–6 times to remove dust and impurities, followed by rinsing with deionized water. The cleaned leaves were shade-dried, ground into a fine powder using a mixer, and subsequently refluxed to obtain the crude extract. The extract was filtered and stored in an airtight container for further use. The resulting plant extract was employed as a plant-derived processing additive during the hydrothermal synthesis of SrMoO_4_:Ho^3+^ nanoparticles. Since detailed phytochemical profiling and independent measurements of surface-active properties were not performed for the specific extract used in this study, no specific phytochemical constituent or surfactant mechanism is assigned to the extract.

#### 2.2.2 Hydrothermal synthesis of Ho^3+^-doped SrMoO_4_

Stoichiometric amounts of Sr(NO_3_)_2_·6H_2_O, Ho(NO_3_)_3_·5H_2_O, and (NH_4_)_6_Mo_7_O_24_·4H_2_O were used for the synthesis. The Chikku leaf extract was employed as a plant-derived processing additive during the hydrothermal process. Initially, Sr(NO_3_)_2_·6H_2_O and Ho(NO_3_)_3_·5H_2_O were dissolved in deionized water and stirred for 10 min. Subsequently, the (NH_4_)_6_Mo_7_O_24_·4H_2_O solution was added dropwise to the above mixture and stirred for 30 min to obtain a uniform solution. The pH was adjusted to 10 using 0.1 N NaOH solution. The resulting mixture was transferred to a Teflon-lined stainless-steel autoclave and maintained at 180 °C for 24 h in a muffle furnace. The product was collected, washed several times with distilled water and ethanol, and dried at 100 °C for 5 h. The dried powder was ground using an agate mortar and pestle, and the final product was stored in airtight vials for further characterization. A schematic representation of the complete synthesis procedure for the SrMoO_4_:Ho^3+^ nanoparticles, including Chikku leaf extract preparation, precursor mixing, pH adjustment, hydrothermal treatment, washing, drying, and grinding, is presented in Fig. 1.

**Fig. 1:**
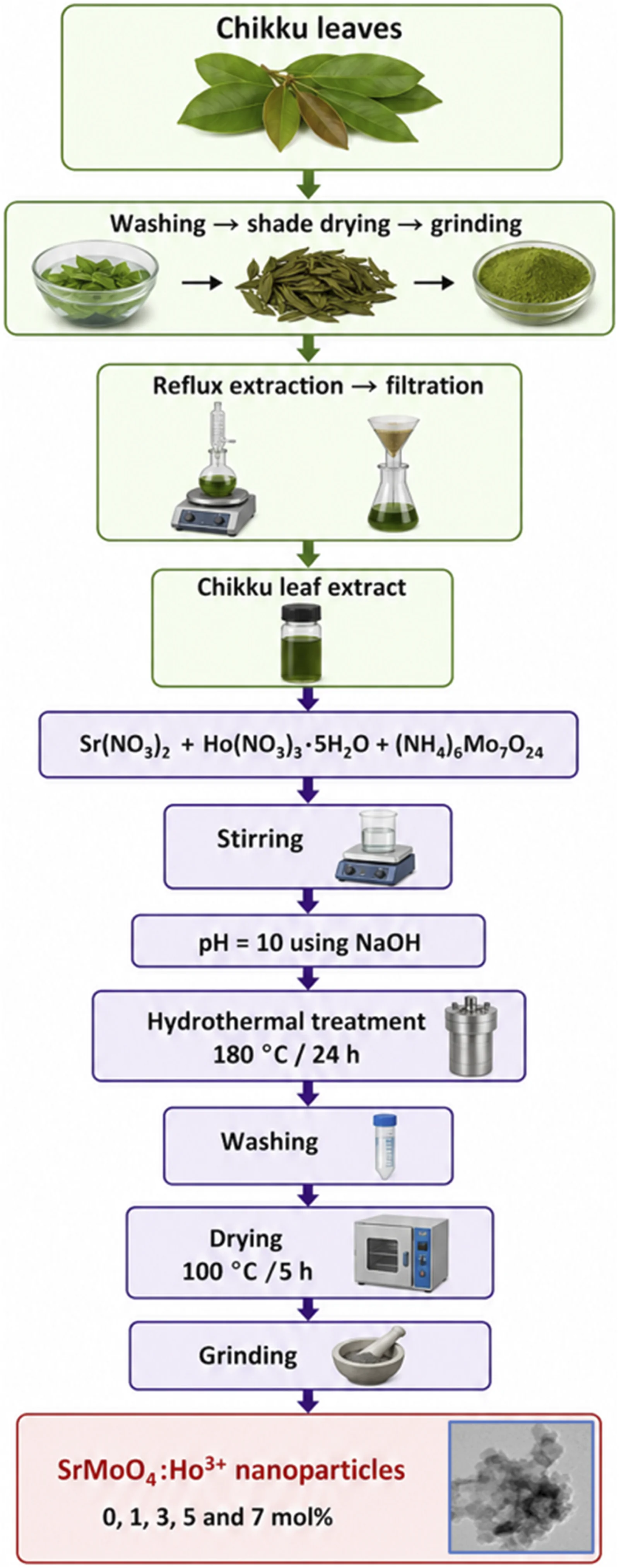
Schematic illustration of the preparation of SrMoO_4_:Ho^3+^ nanoparticles using Chikku (*Manilkara zapota*) leaf extract-assisted hydrothermal synthesis.

## 3. Characterization

The synthesized samples were systematically characterized using a combination of analytical techniques. X-ray diffraction (XRD, Rigaku Miniflex, Cu-Kα radiation, 40 kV/15 mA) was employed to determine the crystalline phase and structural parameters. Fourier-transform infrared spectroscopy (FTIR, Shimadzu IRSpirit, transmittance mode, spectral range 4000–400 cm^−1^) was used to identify functional groups and confirm phase purity. UV-visible diffuse reflectance spectroscopy (DRS, Shimadzu UV-2600i) was performed to evaluate the optical absorption properties and band gap energy. The surface morphology and particle size distribution were examined using field emission scanning electron microscopy (FE-SEM) and transmission electron microscopy (TEM, FEI Tecnai G2S). Photoluminescence (PL) studies, emission, excitation, including lifetime and quantum yield measurements, were conducted using an FLS1000 Edinburgh Instrument. The excitation sources comprised a 450 W Xenon arc lamp (250–900 nm) and a 200 mW He–Cd laser (325 nm).

## 4. Results and discussions

### 4.1. Phase analysis

The crystallographic features of the synthesized SrMoO_4_:Ho^3+^ (1–7 mol%) nanoparticles were examined by PXRD, and the corresponding patterns are shown in Fig. 2. The diffraction profiles of sample reveal sharp and intense reflections, which are well indexed to the tetragonal scheelite-type structure (*I*4_1_/*a* space group), in excellent agreement with the standard JCPDS card no. 08-0482. The absence of extraneous reflections in all compositions indicates that the hydrothermal route, assisted by Chikku leaf extract, yields single-phase products with high crystallinity. Notably, the introduction of Ho^3+^ ions does not result in an observable change in the fundamental scheelite structure within the resolution of the present PXRD measurements. The absence of detectable additional crystalline phases, together with the observed variation in the diffraction features with increasing Ho^3+^ concentration, is consistent with Ho^3+^ incorporation into the SrMoO_4_ host lattice. PXRD alone cannot conclusively establish the substitutional site or local chemical environment of Ho^3+^ ions. However, careful inspection of the diffraction profiles reveals systematic broadening of the Bragg peaks with increasing Ho^3+^ concentration, particularly for the (1 1 2) and (2 2 0) reflections. This line broadening can be correlated with a reduction in crystallite size and the accumulation of microstrain within the lattice. The average crystallite size was quantified using Debye–Scherrer's relation,^45^ and the results are presented in Table 1. The size decreases from 34 nm for the pristine sample to 25 nm for the 7 mol% Ho^3+^ sample, indicating a concentration-dependent reduction in the average coherent crystallite size. These values represent the average coherent crystallite size obtained from PXRD and therefore should not be directly equated with the physical particle dimensions observed by electron microscopy. The observed reduction in crystallite size may be associated with concentration-dependent changes in nucleation and crystal-growth processes. The substantial difference between the ionic radii of Sr^2+^ (1.26 Å, CN = 8) and Ho^3+^ (0.901 Å, CN = 8) provides a plausible basis for local structural perturbations if Ho^3+^ is incorporated into the SrMoO_4_ lattice. Similar rare-earth-induced size confinement effects have been reported in scheelite-type molybdates and tungstates,^46^ where dopant incorporation modifies surface energy anisotropy and alters the thermodynamics of crystallite growth. The incorporation of trivalent Ho^3+^ at divalent Sr^2+^ sites, if substitutional, would require local charge compensation. However, the present PXRD data do not provide direct evidence for the identity or concentration of the compensating defects. Therefore, specific defect species such as cation vacancies or oxygen interstitials cannot be assigned from the present measurements alone. The observed reduction in crystallite size and peak broadening indicate changes in the structural characteristics of the samples that may influence their optical behavior. However, the present PXRD measurements alone cannot establish whether specific defect states or defect-mediated strain are responsible for the observed changes in the optical properties.

**Fig. 2:**
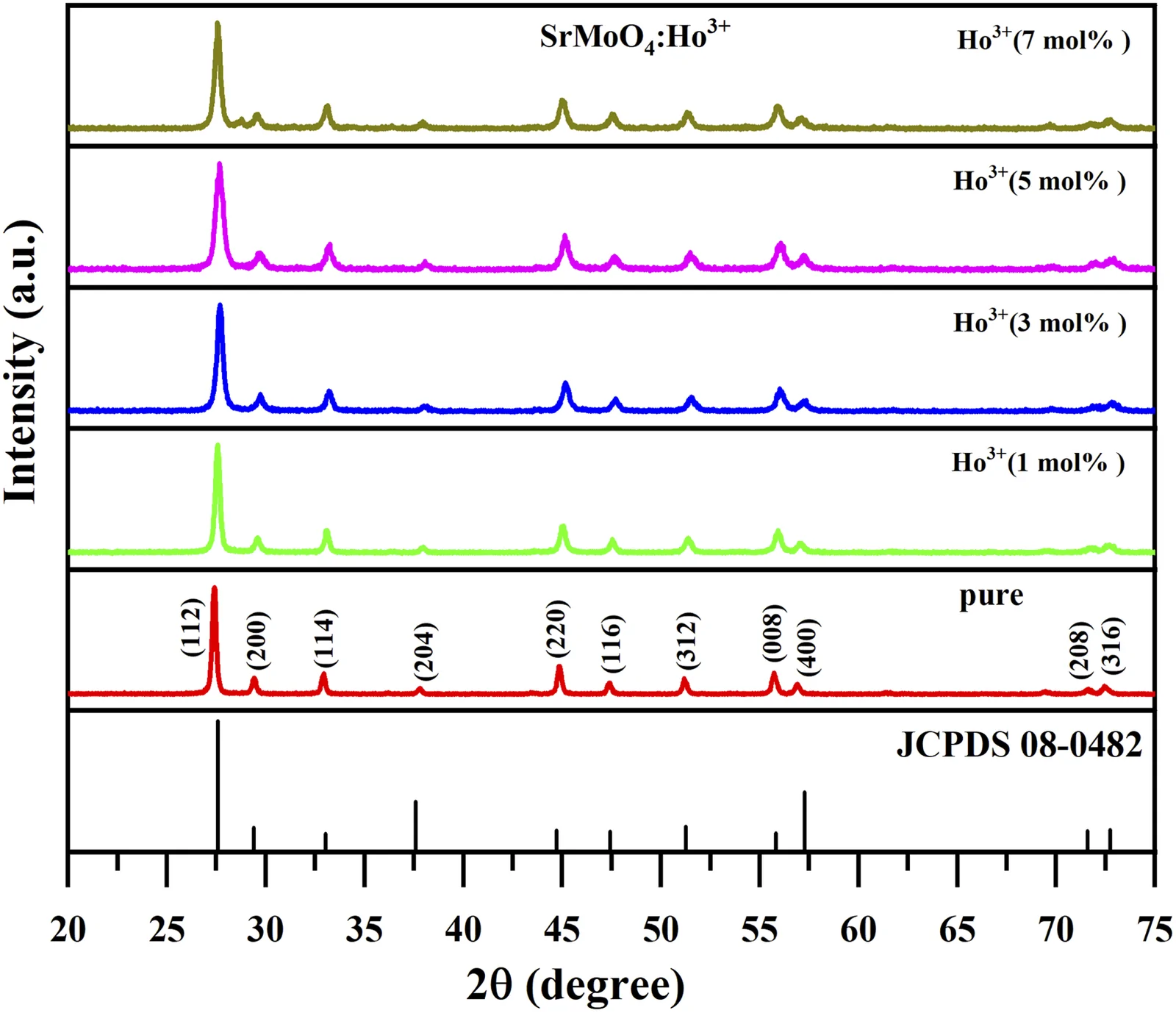
PXRD patterns of pure and SrMoO_4_:Ho^3+^ (1–7 mol%) doped nanoparticles.

**Table 1:** Estimated crystallite size and band gap values of pure and SrMoO_4_:Ho^3+^(1–7 mol%) nanoparticles

Samples	Average crystallite size(nm) Debye–Scherrer's method (d)	Band gap (eV)
Pure	34	4.10
Ho^3+^ (1 mol%)	31	4.12
Ho^3+^ (3 mol%)	29	4.14
Ho^3+^ (5 mol%)	27	4.15
Ho^3+^ (7 mol%)	25	4.17

The optical absorption behavior of pristine and Ho^3+^-doped SrMoO_4_ nanoparticles were investigated using UV-vis diffuse reflectance spectroscopy, and the corresponding spectra are shown in Fig. 3(a). Pristine SrMoO_4_ exhibits a pronounced absorption edge in the ultraviolet region, consistent with its wide-band-gap nature. With increasing Ho^3+^ concentration, a gradual broadening and modification of the absorption-edge profile is observed rather than a distinct shift of a well-defined absorption peak. The corresponding optical band-gap energies were therefore estimated from the Tauc plots shown in Fig. 3(b).

**Fig. 3:**
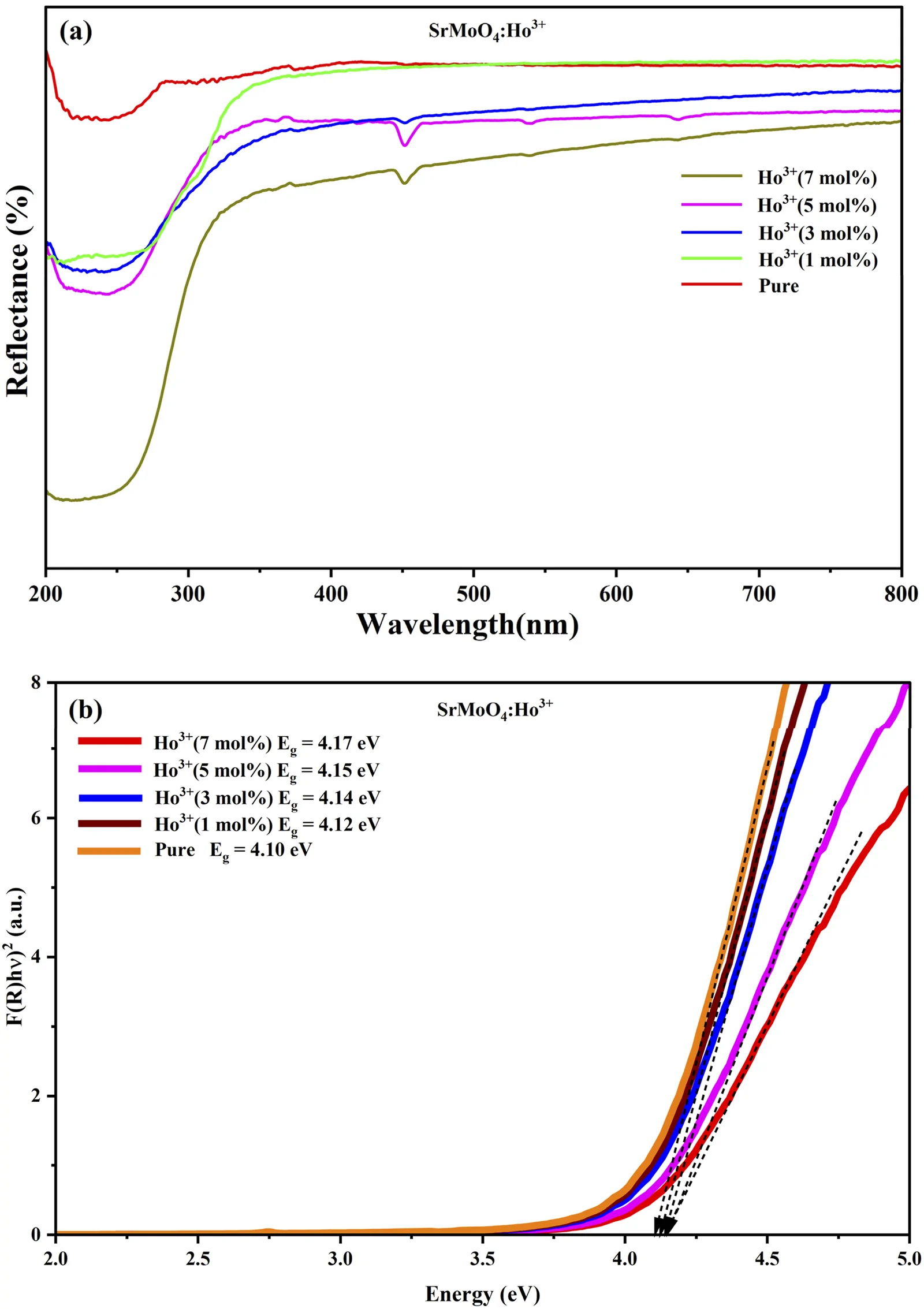
(a) UV-vis diffuse reflectance spectra and (b) Tauc plots of pristine and Ho^3+^-doped SrMoO_4_ nanoparticles (1–7 mol% Ho^3+^). The dashed black lines represent the linear fitting used to estimate the optical band-gap energies.

The Tauc plots were constructed using (*F*(*R*)*hν*)^2^*versus* photon energy (*hν*),^47–49^ as shown in Fig. 3(b). The dashed black lines represent the linear fitting and extrapolation of the appropriate linear regions of the Tauc plots to the energy axis for estimation of the optical band-gap energy. The band-gap energy was estimated to be 4.10 eV for pure SrMoO_4_ and 4.17 eV for 7 mol% Ho^3+^ doped SrMoO_4_, with the corresponding values listed in Table 1. Although a gradual increase in the estimated optical band-gap energy from 4.10 to 4.17 eV is observed with increasing Ho^3+^ concentration, this relatively small variation is interpreted cautiously. Considering that the crystallite sizes remain in the range of approximately 25–34 nm, a pronounced quantum-confinement effect is not considered sufficient to explain the observed band-gap variation. The slight increase in the estimated optical band gap may instead reflect concentration-dependent modifications of the local structural and electronic environment associated with Ho^3+^ incorporation. However, the present DRS measurements do not provide sufficient evidence to distinguish among possible contributions from local structural modification, strain, defect-related effects, or Ho^3+^-induced electronic states. Therefore, no single mechanism is assigned as the definitive origin of the observed band-gap variation.

The FTIR spectra of pure and Ho^3+^ doped SrMoO_4_ nanoparticles are shown in Fig. 4. The spectra reveal distinct vibrational features associated with the scheelite-type tetragonal structure, as well as additional signals linked to surface-adsorbed species and dopant induced modifications. A broad absorption band centered around 3426 cm^−1^ is observed in all samples, attributes to the O–H stretching vibration of physisorbed water molecules and hydroxyl groups on the nanoparticle surface. The corresponding bending mode of molecular water appears near 1636 cm^−1^. These surface hydroxyls are common in hydrothermally prepared oxides and can play a dual role, they serve as charge trapping sites and also facilitate photocatalytic reactions by generating surface radicals under UV irradiation. The sharp vibrational band detected at 1396 cm^−1^ is assigned to the asymmetric stretching vibrations of residual nitrate (NO_3_^−^) groups originates from incomplete decomposition of precursor salts during the synthesis. The presence of this feature is less pronounced in highly doped samples, suggesting increased Ho^3+^ incorporation enhances the lattice stabilization and reduces residual anionic impurities.^50^ The most prominent bands are located in the low wavenumber region, representing the metal–oxygen bonding within the scheelite lattice. The strong absorption near 810 cm^−1^ is attributed to the symmetric stretching vibrations of Mo–O bonds in [MoO_4_]^2−^ tetrahedra, while the feature around 745 cm^−1^ corresponds to asymmetric stretching modes. The band at 411 cm^−1^ is assigned to bending vibrations of O–Mo–O linkages. Together, these peaks confirm the formation of well-defined MoO_4_ tetrahedra, which are the fundamental building blocks of the SrMoO_4_ lattice.

**Fig. 4:**
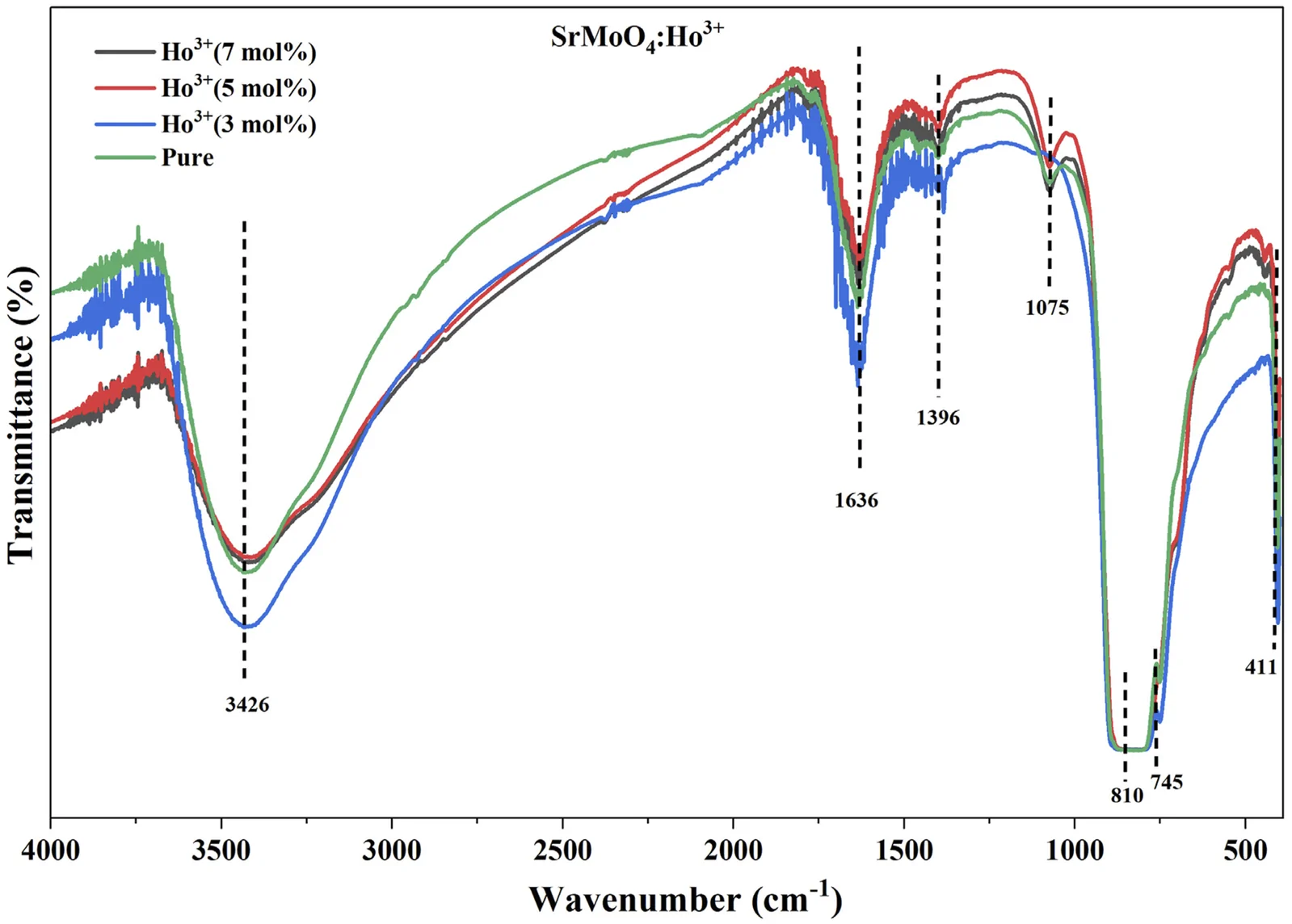
FTIR spectra of pure and SrMoO_4_:Ho^3+^ (3,5 and 7 mol%) doped nanoparticles.

### 4.2. Morphology analysis

The surface morphology of pure and Ho^3+^ doped SrMoO_4_ (7 mol%) nanoparticles was investigated by FESEM, and the representative images are shown in Fig. 5(a and b). The pure SrMoO_4_ sample predominantly exhibits agglomerated quasi-spherical particles. With 7 mol% Ho^3+^ concentration, the morphology becomes increasingly anisotropic, with elongated rod-like features forming dense aggregated structures. This concentration associated morphological evolution indicates that Ho^3+^ concentration influences the nucleation and crystal growth processes during hydrothermal synthesis. However, the specific mechanism responsible for the observed morphological changes cannot be established from FESEM observations alone. The observed morphological changes are based on the FESEM images obtained for the Ho^3+^ concentration and represent the apparent surface morphology at the FESEM observation scale. Because HRTEM characterization was performed only for the 7 mol% Ho^3+^ sample, the present TEM dataset does not independently verify the morphology of the primary nanoparticles at each Ho^3+^ concentration. Therefore, the concentration-associated morphological evolution discussed here is based primarily on the FESEM observations. It should be noted that the FESEM images are presented primarily to compare the apparent surface morphology and aggregation behavior as a function of Ho^3+^ concentration. Quantitative particle-size distributions were not independently determined from the FESEM images for each composition. Therefore, the FESEM images are not used to assign concentration-dependent particle-size values. The quantitative particle-size analysis presented in this work is based on the HRTEM image of the 7 mol% Ho^3+^ sample, for which the observed particle dimensions are predominantly in the range of 40–70 nm, with a mean particle size of approximately 55 nm. The chemical composition of the samples was further confirmed by energy-dispersive X-ray spectroscopy (EDX), as shown in Fig. 5(c and d). For the pure SrMoO_4_, only Sr, Mo, and O elements were detected, confirming phase purity without extraneous contaminants. In the 7 mol% Ho^3+^ doped samples, distinct Ho signals appear, confirming successful incorporation of Ho^3+^ into the lattice.

**Fig. 5:**
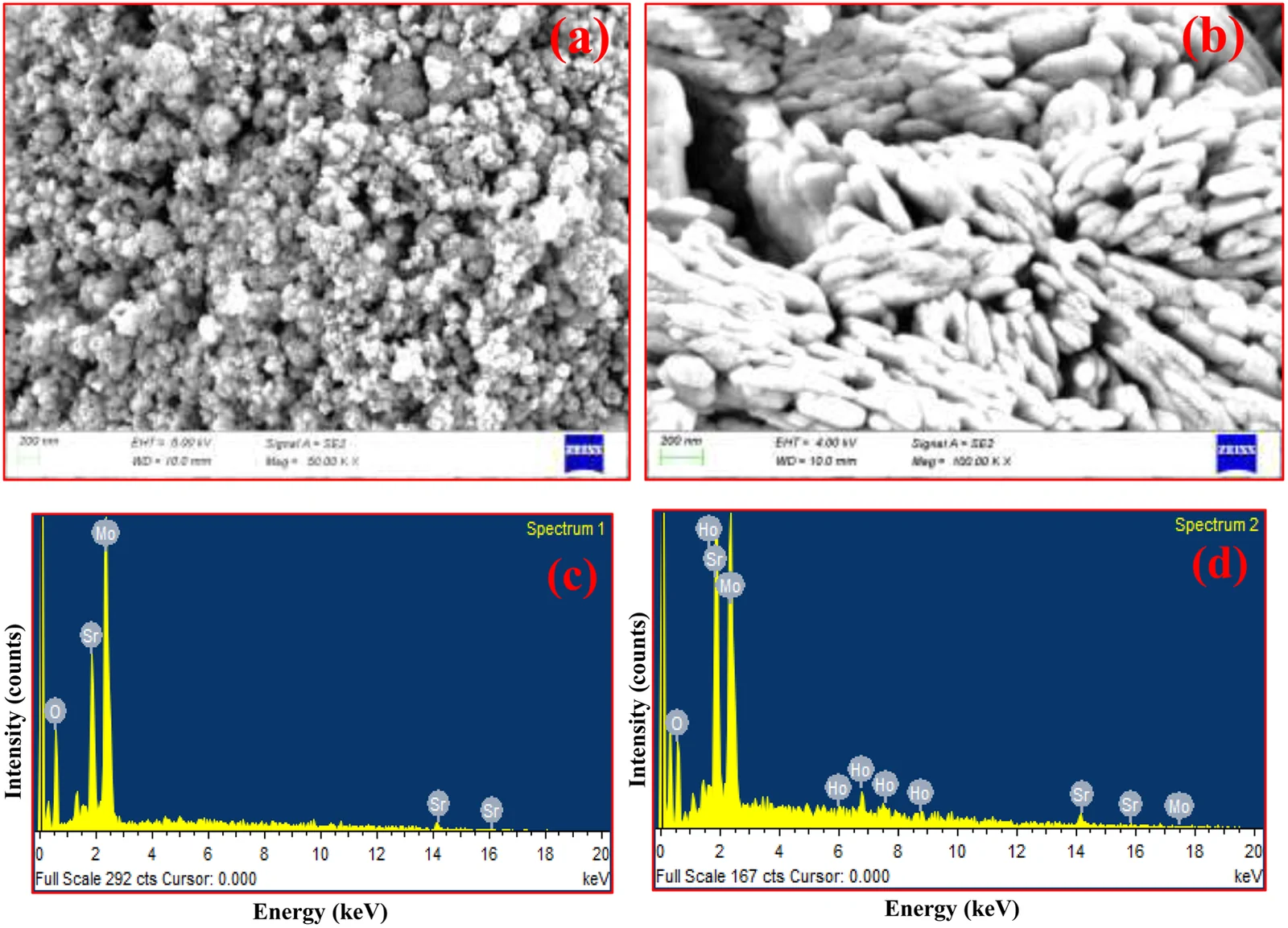
(a and b) FESEM images and (c and d) EDAX spectrum of pure and SrMoO_4_:Ho^3+^ (7 mol%) doped nanoparticles.

### 4.3. HRTEM analysis

The high-resolution transmission electron microscopy (HRTEM) images of SrMoO_4_:Ho^3+^ (7 mol%) nanoparticles are presented in Fig. 6(a and b). The HRTEM images reveal predominantly quasi-spherical primary nanoparticles with a tendency toward agglomeration. The apparent morphology observed by HRTEM differs from the larger anisotropic features observed in the FESEM images, particularly at higher Ho^3+^ concentrations. This difference arise from the different observation scales and imaging characteristics of the two techniques: FESEM primarily provides information on the surface morphology and larger particle assemblies, whereas HRTEM provides high-resolution information on individual electron-transparent nanoparticles. However, because HRTEM characterization was performed only for the 7 mol% Ho^3+^ sample, this observation should not be generalized to the other Ho^3+^ concentrations. The concentration-associated morphological evolution discussed in this work is therefore based primarily on the FESEM observations.

**Fig. 6:**
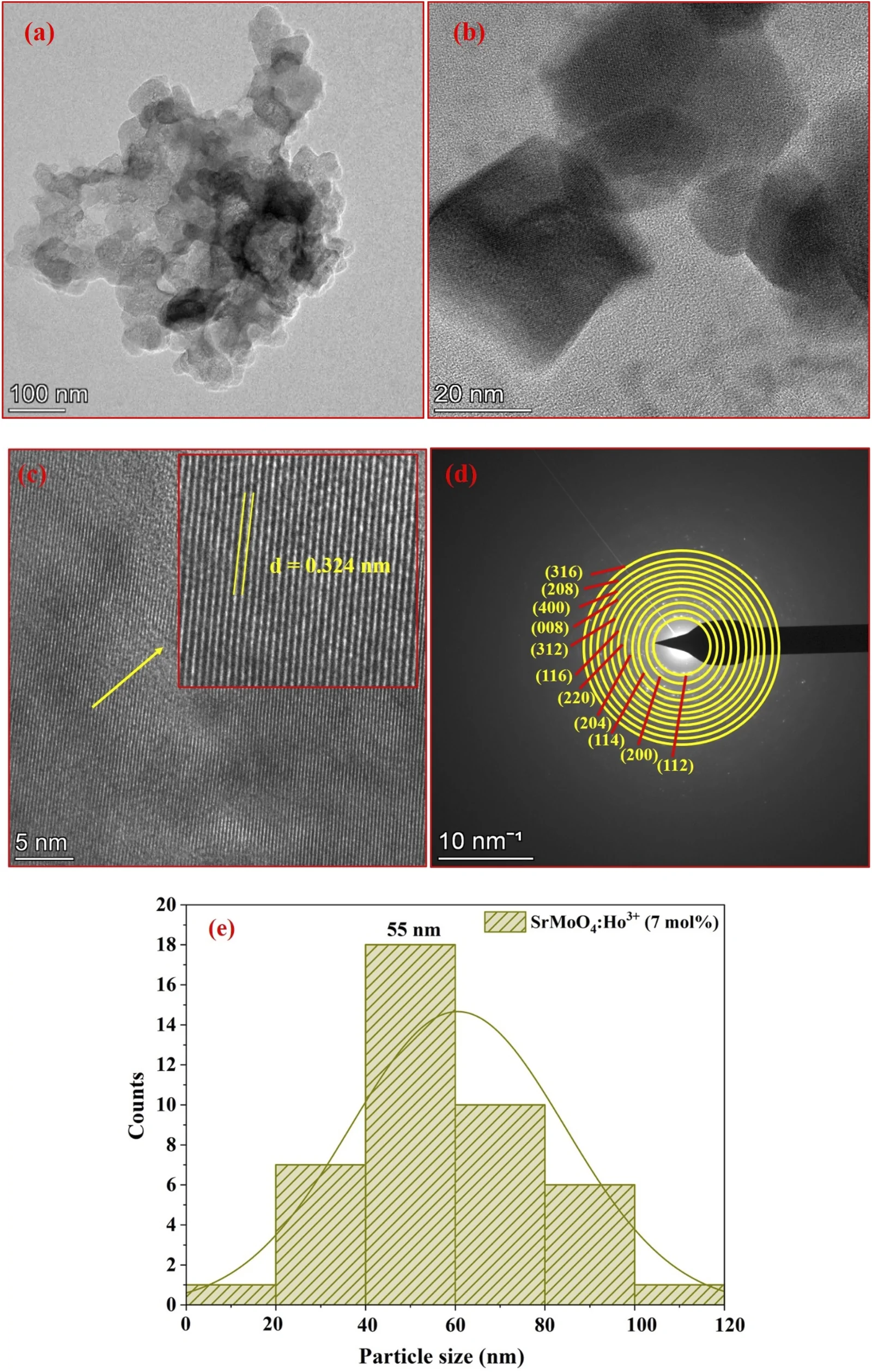
HRTEM images and SAED patterns of SrMoO_4_:Ho^3+^(7 mol%) nanoparticles: (a and b) HRTEM images of SrMoO_4_:Ho^3+^(7 mol%) (c) lattice fringes showing an interplanar distance (d) SAED pattern of SrMoO_4_:Ho^3+^(7 mol%), (e) particle size distribution of SrMoO_4_:Ho^3+^(7 mol%) nanoparticles.

The primary nanoparticles observed in the HRTEM images have particle dimensions predominantly in the range of 40–70 nm, with a mean particle size of approximately 55 nm, as obtained from the particle-size distribution shown in Fig. 6(e). The magnified lattice-resolved image [Fig. 6(c)] reveals distinct and periodic lattice fringes, demonstrating the high crystallinity of the sample. The measured interplanar spacing of 0.324 nm corresponds to the (1 1 2) crystallographic planes of tetragonal scheelite-type SrMoO_4_, in agreement with the PXRD analysis. The clear visibility of the lattice fringes indicates well-defined local crystallinity at the examined region.

The selected-area electron diffraction (SAED) pattern [Fig. 6(d)] exhibits concentric diffraction rings with bright diffraction spots. The observed rings can be indexed to the (1 1 2), (2 0 0), (2 2 0), (3 1 2), (4 0 0), and (3 1 6) planes of tetragonal SrMoO_4_, consistent with the PXRD results. The diffraction features indicate the crystalline nature of the sample. The absence of additional diffraction rings within the resolution of the present SAED measurement supports the formation of the SrMoO_4_ crystalline phase without detectable secondary crystalline phases.

The particle-size distribution analysis [Fig. 6(e)] shows that the majority of the observed nanoparticles fall within the 40–70 nm range, with an average particle size of approximately 55 nm. This value is larger than the crystallite size of approximately 25 nm estimated from PXRD. The difference is expected because PXRD provides an estimate of the average coherent diffraction domain size, whereas HRTEM measures the physical dimensions of the nanoparticles or particle assemblies visible in the micrograph. Consequently, an individual nanoparticle observed by HRTEM may contain multiple coherently or partially coherently diffracting domains. It should be noted that comprehensive HRTEM characterization and particle-size statistics for all Ho^3+^ concentrations would provide further independent verification of the concentration-associated morphological evolution. Such concentration-resolved HRTEM measurements were not performed in the present study; therefore, the HRTEM results are used primarily to characterize the morphology, crystallinity, and particle dimensions of the 7 mol% Ho^3+^ sample.

## 5. Analysis of luminescence properties

Fig. 7(a) shows the excitation spectra of SrMoO_4_:Ho^3+^ (7 mol%) nanoparticles, monitored at the dominant emission wavelength of 657 nm. The excitation profile reveals a strong and sharp absorption band at 451 nm, which is assigned to the ^5^I_8_ → ^5^G_6_ transition of Ho^3+^ ions.^51^ The intensity and narrow linewidth of this feature confirm its origin from the intra-4f transitions rather than host-related charge transfer processes, which usually manifest as broad bands in the UV region. The observation of such a pronounced excitation band indicates the successful incorporation of Ho^3+^ ions into the SrMoO_4_ host lattice, where the local crystal field enhances the oscillator strength by relaxing the Laporte-forbidden selection rules. This efficient excitation at 451 nm, which lies in the blue spectral region, makes the phosphor highly compatible with GaN-based blue-LED chips, a critical requirement for solid-state lighting applications.^52,53^

**Fig. 7:**
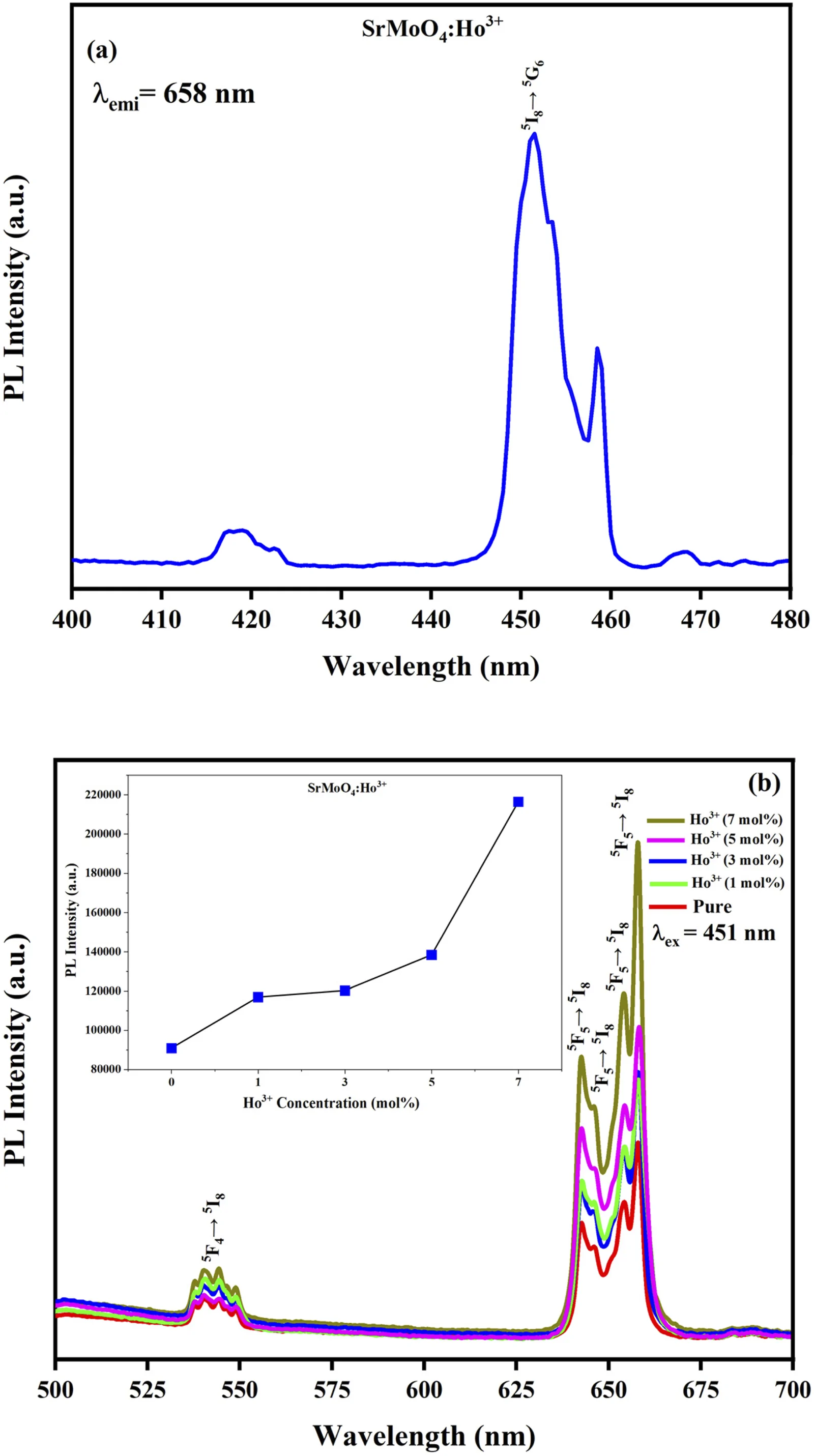
(a) Excitation and (b) emission spectra of pure and SrMoO_4_:Ho^3+^ (1–7 mol%) doped nanoparticles.

Fig. 7(b) shows emission spectra of pure and SrMoO_4_:Ho^3+^ nanoparticles under 451 nm excitation as a function of dopant concentration (1–7 mol%). The emission spectra are characterized by two distinct sets of sharp lines: (i) the green emission band centered at 537–548 nm, corresponding to the ^5^S_2_/^5^F_4_ → ^5^I_8_ transitions, and (ii) the red emission band in the 642–657 nm range, assigned to the ^5^F_5_ → ^5^I_8_ transition of Ho^3+^ ions. The presence of multiple Stark components in both emission regions reflects the splitting of Ho^3+^ multiplets under the low-symmetry crystal field of the tetragonal scheelite SrMoO_4_ structure. Among these transitions, the red emission in the ∼642–657 nm region exhibits the highest intensity under the present excitation conditions. This observation indicates that the red-emission channel is dominant in the measured photoluminescence spectrum; however, the relative emission intensity alone does not provide a quantitative measure of the radiative efficiency or fluorescence quantum efficiency of the material. Therefore, no quantitative claim regarding enhanced radiative decay is made in the present study.^54,55^

The inset of Fig. 7(b) shows the variation of emission intensity with Ho^3+^ concentration. A systematic increase in luminescence intensity is observed from 1 mol% to 7 mol% doping, which is attributed to the increased number of optically active Ho^3+^ centers contributing to radiative recombination. The monotonic growth in intensity without noticeable quenching indicates that cross-relaxation processes, such as ^5^S_2_ + ^5^I_8_ → ^5^F_4_ + ^5^I_6_, remain insignificant within this doping range. This suggests that 7 mol% is close to the optimal concentration for maximizing output intensity, beyond which concentration quenching is likely to dominate due to enhanced non-radiative energy migration among Ho^3+^ ions.^56–58^

The emission mechanism can be described as follows: upon excitation at 451 nm, Ho^3+^ ions are promoted to the ^5^G_6_ state. This is followed by rapid non-radiative relaxation to the ^5^S_2_/^5^F_4_ and subsequently to the ^5^F_5_ manifold through host-assisted multiphonon processes, favored by the vibrational modes of the Mo–O polyhedra (∼800–900 cm^−1^). Radiative relaxation from these levels gives rise to the characteristic green and red emissions. The higher relative intensity of the ^5^F_5_ → ^5^I_8_ transition results in a strongly red-weighted emission spectrum under the present excitation conditions; however, the relative intensity does not by itself establish the quantitative efficiency of the underlying relaxation pathways. The proposed excitation and relaxation pathways of Ho^3+^ ions in the SrMoO_4_ host are schematically illustrated in Fig. 8. The diagram summarizes the excitation at 451 nm, subsequent non-radiative relaxation, and the characteristic green and red radiative transitions observed in the PL spectra.

**Fig. 8:**
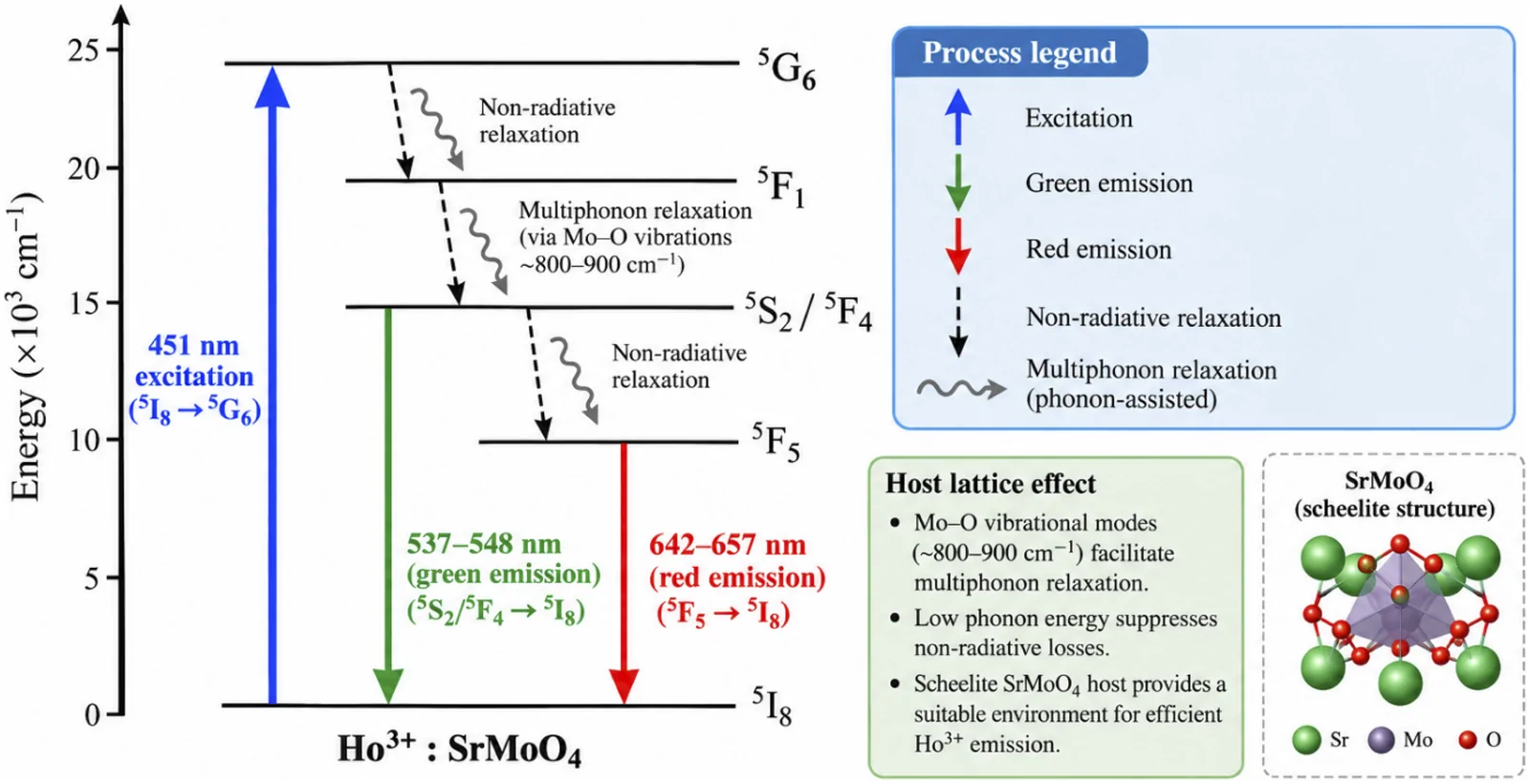
Schematic energy-level diagram illustrating the proposed photoluminescence emission mechanism of Ho^3+^ ions in SrMoO_4_ under 451 nm excitation, including the non-radiative relaxation pathways and green (537–548 nm) and red (642–657 nm) emission transitions.

Fig. 9. Shows the time-resolved photoluminescence characteristics of pure and Ho^3+^-activated SrMoO_4_ phosphors were investigated to evaluate the recombination dynamics of excited carriers and to assess the effect of activator concentration on emission persistence. The decay profiles were recorded under an excitation wavelength of 451 nm, while monitoring emission at 658 nm, corresponding to the characteristic red transition of Ho^3+^ ions. The recorded decay curves exhibit a gradual decrease in emission intensity with increasing delay time, indicating the relaxation of excited states through both radiative and non-radiative recombination processes within the host lattice. The variation of luminescence intensity was analyzed using a standard exponential decay model commonly applied to rare-earth activated systems:

1 *I*(*t*) = *I*_0_ × *e*^*−t*/*τ*^

**Fig. 9:**
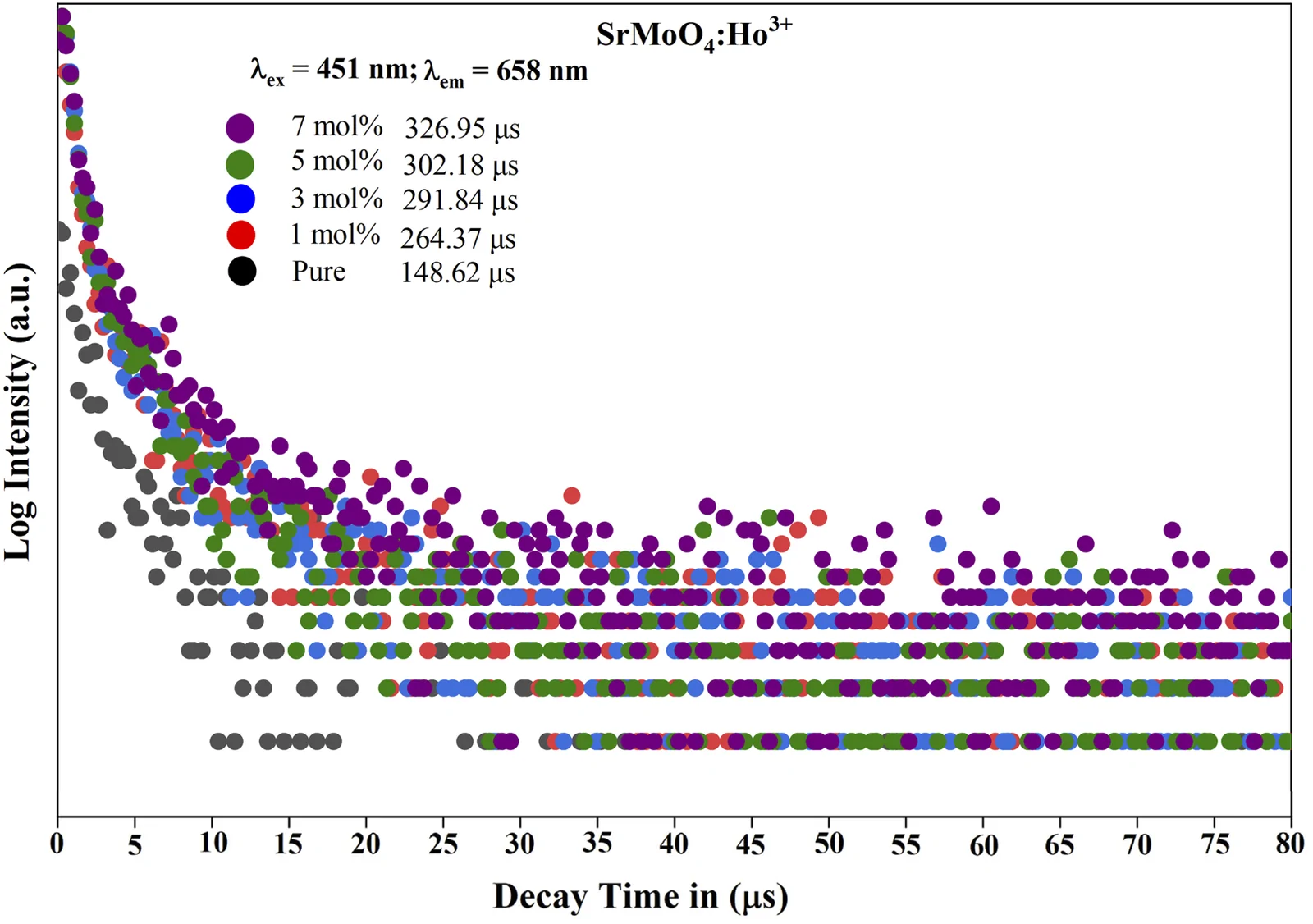
Photoluminescence (PL) decay curves of pure and SrMoO_4_:Ho^3+^ (1–7 mol%) doped nanoparticles.

In this relation, *I*(*t*) represents the emission intensity at time *t*, *I*_0_ denotes the initial intensity immediately after excitation, and *τ* corresponds to the decay lifetime of the emitting state.^59^ The lifetime parameter represents the characteristic decay time of the emitting state and provides information on the emission decay dynamics of the material. The decay curves reveal a clear dependence of emission lifetime on Ho^3+^ concentration. The undoped SrMoO_4_ sample exhibits the shortest lifetime, indicating relatively rapid recombination of excited carriers in the absence of activator ions. Upon introduction of Ho^3+^ ions into the lattice, a significant increase in emission lifetime is observed, confirming the role of rare-earth ions as effective luminescent centers capable of capturing excitation energy and releasing it gradually through radiative transitions.

The average lifetime values obtained from the PL decay curves are 148.62, 264.37, 291.84, 302.18 and 326.95 µs for pristine, 1, 3, 5 and 7 mol% Ho^3+^ doped SrMoO_4_, respectively (Table 2). A progressive increase in lifetime is observed with increasing Ho^3+^ concentration, indicating a concentration-dependent modification of the emission decay dynamics. The longer decay time observed for the Ho^3+^ doped samples indicates a change in the emission decay behavior following Ho^3+^ activation. No decrease in lifetime is observed up to 7 mol% Ho^3+^, suggesting that significant concentration quenching is not evident within the investigated concentration range. Thus, the lifetime results demonstrate that Ho^3+^ concentration provides an effective means of tuning the emission decay characteristics of SrMoO_4_.

**Table 2:** The calculated average lifetimes for the investigated compositions

Composition	Lifetime (µs)
Pure SrMoO_4_	148.62
1 mol% Ho^3+^	264.37
3 mol% Ho^3+^	291.84
5 mol% Ho^3+^	302.18
7 mol% Ho^3+^	326.95

The CIE chromaticity coordinates of Ho^3+^ doped SrMoO_4_ nanoparticles are shown in the CIE 1931 *x*–*y* chromaticity diagram in Fig. 10. The diagram represents the standard CIE 1931 chromaticity space, where the horseshoe shaped boundary corresponds to the spectral locus and the straight boundary represents the line of purples. The plotted coordinates indicate the perceived colour characteristics of the photoluminescence emission from the investigated samples. Further, examined using the CIE 1931 chromaticity diagram and correlated color temperature (CCT) values.^60,61^ The undoped SrMoO_4_ sample exhibits chromaticity coordinates (*x* = 0.468, *y* = 0.506), corresponding to a warm white emission (CCT = 3230 K) with a slight pinkish tint. The chromaticity of the undoped SrMoO_4_ sample include contributions from host-related defect states and intrinsic luminescence, upon incorporation of Ho^3+^ ions, the chromaticity coordinates undergo a systematic shift along the Planckian locus toward the reddish-orange region. This shift directly reflects the increasing dominance of the ^5^F_5_ → ^5^I_8_ transition (red emission) over the ^5^S_2_/^5^F_4_ → ^5^I_8_ transitions (green emission) with increasing dopant concentration. At lower concentrations (1–5 mol%), the green and red channels coexist with comparable intensity, resulting in chromaticity coordinates positioned in the yellowish-orange region. With further increase in Ho^3+^ loading, the relative contribution of the red ^5^F_5_ → ^5^I_8_ emission becomes more pronounced, resulting in a corresponding change in the chromaticity coordinates. This drives the emission chromaticity deeper into the reddish domain, accompanied by CCT values shifting from 3383 K to 3665 K and the photometric values such as CIE and CCT were depicted and tabulated in Table 3. Such values fall well within the “warm white” region (CCT < 4000 K) as defined by ANSI/IES lighting standards, making the material highly attractive for indoor illumination applications that require comfort-oriented light sources. It is noteworthy that the CCT evolution is not merely a photometric descriptor but directly correlates with the microscopic emission dynamics. The increase in CCT with concentration reflects a spectral redistribution of photon flux from the green channel to the red channel, resulting in warmer perceived light. Simultaneously, the narrowing of emission lines due to the 4f nature of Ho^3+^ ensures high color stability against thermal fluctuations, an advantage over broadband phosphors. Additionally, the CIE coordinates of SrMoO_4_:Ho^3+^ (7 mol%) are located near the blackbody locus, implying high spectral resemblance to natural warm illumination.

**Fig. 10:**
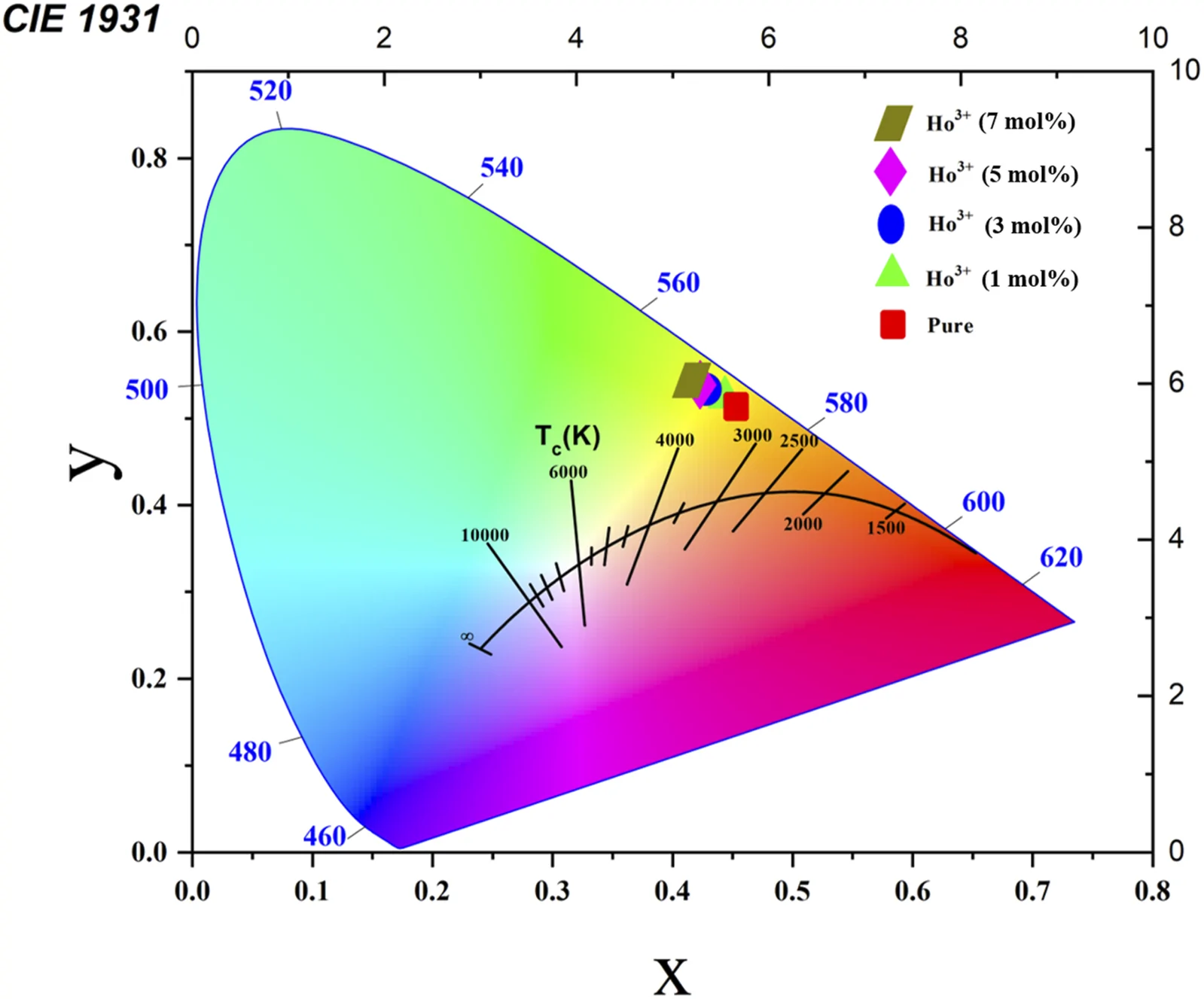
CIE 1931 *x*–*y* chromaticity diagram showing the chromaticity coordinates of SrMoO_4_:Ho^3+^ (1–7 mol%) doped nanoparticles.

**Table 3:** Photometric values of pure and doped nanoparticles

Sample	*X*	*Y*	CCT (K)
Pure	0.468	0.506	3230
Ho^3+^ (1 mol%)	0.459	0.511	3383
Ho^3+^ (3 mol%)	0.441	0.528	3715
Ho^3+^ (5 mol%)	0.446	0.525	3626
Ho^3+^ (7 mol%)	0.444	0.527	3665

## 6. Conclusion

In summary, Ho^3+^ activated SrMoO_4_ nanoparticles were successfully synthesized through a controlled hydrothermal process using Chikku (*Manilkara zapota*) leaf extract as a plant-derived processing additive. PXRD analysis revealed a phase-pure tetragonal scheelite structure for all investigated compositions, with no detectable secondary crystalline phases. The observed structural changes are consistent with Ho^3+^ incorporation into the SrMoO_4_ host lattice; however, direct confirmation of the substitutional site and local chemical environment requires complementary characterization. The average crystallite size decreased from approximately 34 to 25 nm with increasing Ho^3+^ concentration, indicating a concentration-dependent variation in the coherent crystallite size. UV-vis diffuse reflectance measurements showed a gradual modification of the absorption-edge profile, with the estimated optical band gap increasing from 4.10 to 4.17 eV. Considering the crystallite-size range, a pronounced quantum-confinement effect is not considered sufficient to explain this variation, and no single mechanism is assigned as the definitive origin of the observed band-gap change. FESEM observations revealed concentration associated changes in the apparent surface morphology, with predominantly quasi-spherical to prolate-spherical features at lower Ho^3+^ concentrations and increasingly anisotropic features at higher concentrations. HRTEM analysis of the 7 mol% sample showed predominantly quasi-spherical primary nanoparticles with particle dimensions of approximately 40–70 nm and a mean particle size of about 55 nm. The photoluminescence spectra exhibited characteristic Ho^3+^ emissions in the visible region, with prominent green and red emission bands near 545 and 658 nm, respectively. The relative emission intensity increased with Ho^3+^ concentration, reaching its highest value for the 7 mol% sample. Time-resolved photoluminescence measurements showed a progressive increase in emission lifetime from 148.62 µs for pristine SrMoO_4_ to 326.95 µs for the 7 mol% Ho^3+^ doped sample. The absence of a decrease in lifetime within the investigated concentration range indicates that significant concentration quenching is not evident up to 7 mol% Ho^3+^. Overall, the results establish a clear concentration-dependent relationship between the structural characteristics, morphology, visible photoluminescence, and emission decay dynamics of Ho^3+^-activated SrMoO_4_ nanoparticles. The observed tunability of the visible emission characteristics and extended emission lifetime suggests that this material system offers promising potential for applications requiring reliable and tunable luminescent performance in solid-state lighting technologies.

## Author contributions

Arpitha H J: writing – original draft, conceptualization and study design, synthesis and experimental methodology, data curation and validation, analysis and interpretation, visualization and figure preparation. Kartik Gopal: writing – review & editing, data curation, validation, formal analysis, conceptualization Sunitha D V: writing – review & editing, data curation, validation, supervision, formal analysis, conceptualization. Kalappa Prashantha: formal analysis and methodology.

## Conflicts of interest

There are no conflicts to declare.

## Data Availability

All data generated and analyzed during this study are included in research article in the form of plots/graphs and tables. No additional raw data are available.
